# Unraveling the Burden of Pancreatic Cancer in the 21st Century: Trends in Incidence, Mortality, Survival, and Key Contributing Factors

**DOI:** 10.3390/cancers17101607

**Published:** 2025-05-09

**Authors:** Jakob Zottl, Christian Günther Sebesta, Elena Tomosel, Marie-Christine Sebesta, Christian Sebesta

**Affiliations:** 1 Science Center Donaustadt, 1220 Vienna, Austria; sebesta.christianjr@gmail.com (C.G.S.); marie.sebesta@gmail.com (M.-C.S.); 2 2nd Medical Departement, Klinik Donaustadt, Science Center Donaustadt, 1220 Vienna, Austria; elena.tomosel@gesundheitsverbund.at; 3 2nd Medical Departement, Klinik Donaustadt, Science Center Donaustadt, Vienna Cancer Center (VCC), 1220 Vienna, Austria; christian.sebesta@gesundheitsverbund.at

**Keywords:** pancreatic cancer, changes incidence, mortality, survival, risk factors, young onset pancreatic cancer

## Abstract

**Background:** PC has become a significant global health challenge, with incidence and mortality rates rising over the past three decades. While traditionally associated with aging, recent data indicate an increasing burden among younger populations. This study aims to analyze global trends in PC incidence and mortality and to identify key contributing factors, particularly modifiable risk factors such as obesity, diabetes, and smoking. **Methods:** Using data from the Global Burden of Disease Study (GBD) 2021, population-based cancer registries globally and nationally, systematic reviews and analysis trends in PC incidence, mortality and survival were analyzed. To assess epidemiological shifts, we utilized previously published annual percentage change (AAPC) values stratified by region, age group, and sex, as reported in the cited literature. Additionally, the influence of modifiable risk factors was evaluated to determine their contribution to rising incidence rates. **Results:** Between 1990 and 2021, the global incidence of PC increased by 8.9%, from 5.47 to 5.96 per 100,000, with the highest rates observed in high-Sociodemographic-Index (SDI) regions (10.00 per 100,000) and the lowest in low-SDI regions (1.59 per 100,000). Significant increases in incidence were noted in several countries, particularly among men in Iceland (AAPC 8.85) and women in Malta (AAPC 6.04). Early-onset PC is becoming more prevalent, especially among younger women. Modifiable risk factors, including obesity, diabetes, and smoking, play a critical role, with excess body weight contributing to 17.9% of PC cases and smoking to 13.9% in the United States (U.S.). **Conclusions:** The rising burden of PC, particularly among younger populations, highlights the need for targeted prevention strategies, early detection efforts, and further research into the underlying mechanisms driving these trends. Addressing modifiable risk factors could be key to mitigating the increasing incidence of this highly lethal cancer.

## Simple Summary

Pancreatic cancer (PC) is becoming an increasing global concern, with rising cases and deaths over the past three decades. This trend is particularly noticeable among younger adults, especially women, suggesting that the disease may be affecting different age groups than before. While aging remains a key factor, lifestyle-related risks such as obesity, diabetes, and smoking are playing a growing role in its development. This study examines global trends in PC and explores the reasons behind its increasing burden. By identifying key risk factors and high-risk populations, these findings could help improve prevention strategies, enhance early detection, and guide future research to reduce the impact of this deadly disease.

## 1. Introduction

PC ranks as the 12th most common cancer worldwide and, due to its poor prognosis, it is the sixth leading cause of cancer-related deaths, contributing to 5% of all cancer fatalities [1]. Although the 5-year relative survival rate of has slightly improved over the past decades due to advances in diagnostic and therapeutic techniques, it remains one of the lowest, standing at 12.8%, compared to other gastrointestinal malignancies [2]. This is primarily due to the fact that PC is often diagnosed at advanced, locally unresectable stages with distant metastases due to the absence of symptoms in patients and the lack of effective diagnostic tools for early detection [3].

In contrast, prostate cancer—while the second most common cancer in men globally with an estimated 1.47 million new cases in 2022—has considerably higher survival rates due to more widespread screening and earlier detection [4]. In the United States alone, around 288,300 new prostate cancer cases were projected for 2023, making it the most frequently diagnosed malignancy in men [4,5]. Globally, the age-standardized incidence rate (ASIR) for prostate cancer increased from 30.5 per 100,000 in 1990 to 37.9 per 100,000 in 2017, with the most pronounced rise observed in high-income countries—a trend likely linked to improved socioeconomic conditions and increased screening uptake [6,7]. The 5-year survival rate for localized disease is nearly 100%, while it drops to around 37% in metastatic stages [4,5]. Despite high survival in early stages, prostate cancer accounts for 11% of male cancer deaths in the U.S., with approximately 34,700 deaths projected in 2023 [5]. Mortality increases with age and tumor grade, and notable disparities persist, with Black men experiencing higher death rates than White men. Current clinical guidelines emphasize the importance of early detection and individualized treatment approaches to improve outcomes [8,9].

While prostate cancer has benefitted from improvements in screening and survival, the outlook for pancreatic cancer—particularly its early-onset form—remains grim. Early-onset pancreatic cancer (EOPC), defined as pancreatic cancer diagnosed at age 50 or younger, is a relatively rare subset of the disease that has been increasingly observed in developed countries, potentially due to changing lifestyle patterns. This form of pancreatic cancer is associated with distinct clinical and molecular characteristics compared to cases diagnosed at older ages [10,11,12]. It accounts for approximately 8–10% of all pancreatic cancer cases when defined as diagnosis at or before the age of 50. However, when a stricter age threshold of ≤45 years is applied, the frequency decreases to around 3% [13,14,15]. Although EOPC is less common than later-onset pancreatic cancer (LOPC), it accounts for a disproportionately high burden of potential years of life lost—estimated at around 25% in the United States and 40% in Europe. While survival among patients with EOPC has shown some improvement over time, the overall prognosis remains poor [16,17]. Understanding the unique epidemiology and clinical profile of EOPC is therefore essential to address its rising burden, particularly among younger populations.

Trends in PC incidence and mortality show a significant increase in most analyzed countries, making it a major public health concern and an escalating challenge for healthcare systems in both industrialized and developing nations [18,19,20]. In light of the simultaneous decline in mortality rates from other gastrointestinal cancers, such as colorectal and stomach cancer, PC has gained greater significance in the public health discourse [1,21]. PC is projected to become the second leading cause of cancer-related mortality in the U.S. by 2040, surpassing colorectal cancer [22]. The aim of this manuscript is to provide insights into the changing trends in PC incidence, mortality, and survival from the late 20th century to the present, as well as the associated factors driving these developments.

## 2. Material and Methods

This narrative review is based on a comprehensive literature search conducted between November 2024 and February 2025 using PubMed, Embase, and Google Scholar. Relevant studies were identified using combinations of keywords such as “pancreatic cancer”, “incidence”, “mortality”, “survival”, “epidemiology”, “risk factors”, “early-onset pancreatic cancer”, “diabetes”, “obesity”, “smoking”, “pancreatitis”, “COVID-19 Pandemic”, “Neoadjuvant Chemotherapy”, “Surgical Techniques and Centralization”, “Survival Outcomes”, and “Multimodal Therapy”. Diabetes, obesity, and smoking were selected as key search terms because they are the most well-established and frequently investigated risk factors for pancreatic cancer. These factors have a strong evidence base and are consistently linked to both disease incidence and mortality, whereas data on other potential contributors remain comparatively sparse or inconclusive. The search focused on original research articles, systematic reviews, meta-analyses, and epidemiological reports published between 1990 and 2024, with an emphasis on population-based data and global cancer registries (e.g., GBD, Surveillance, Epidemiology, and End Results (SEER), Global Cancer Statistics (GLOBOCAN), and Cancer Research United Kingdom (UK)). Additional grey literature from institutions such as the World Health Organization (WHO), Centers for Disease Control and Prevention (CDC), and the International Agency for Research on Cancer (IARC) was reviewed to support global and regional comparisons. Articles were screened by title and abstract, and those deemed relevant were analyzed in full. Particular attention was given to studies reporting age-standardized incidence and mortality rates (ASIR, ASMR), annual percentage changes (APC, AAPC), and those evaluating modifiable risk factors and demographic trends associated with pancreatic cancer. Data were synthesized narratively to identify and discuss temporal patterns, regional differences, and the impact of epidemiological shifts on pancreatic cancer burden worldwide.

### Epidemiology of PC

According to GLOBOCAN 2022, there were 510,566 new cases of PC, accounting for 2.6% of all cancer diagnoses globally in 2022, as well as 467,005 deaths, representing 4.8% of all cancer-related fatalities [1]. By sex, there were 269,583 new cases in men, with an age-standardized rate (ASR) of 5.5% and a cumulative risk of 0.64%, and 240,983 new cases in women, with an ASR of 4% and a cumulative risk of 0.44%. Region-specific incidence rates showed the highest ASRs in Western Europe (10.1% for males, 7.7% for females), Eastern Europe (9.8% for males, 5.9% for females), Northern America (9.6% for males, 7.4% for females), and Southern Europe (8.8% for males, 6.7% for females). Among EU countries, the highest incidence rates in 2020 were observed in Hungary (11.2/100,000), Slovakia (9.6/100,000), and the Czech Republic (9.5/100,000) [23]. The lowest incidence rates were found in South Central Asia (1.5% for males, 0.9% for females), Central Africa (1.9% for males, 1.2% for females), Eastern Africa (1.9% for males, 1.6% for females), and Western Africa (2.0% for males, 1.5% for females) [1].

The data suggest an association between the Human Development Index (HDI)—a measure of average achievements in health, education, income, and life expectancy [24]—and the incidence of PC, with the highest incidence found in countries with a very high HDI, compared to those with a high, low, or medium HDI [25]. A population-based systematic analysis of data from GLOBOCAN 2020 by Wang et al. (2024) showed that countries with higher levels of HDI have a higher lifetime risk of developing gastrointestinal cancers [26]. High-HDI countries also exhibit the highest death rates, particularly in the third HDI quartile. The lifetime risk of developing PC is 1.72% in very-high-HDI countries, compared to 0.82% in high-HDI countries, while the risk of dying from PC is 1.60% in very-high-HDI countries and 0.81% in high-HDI countries. A region-specific analysis revealed that Western Europe has the highest lifetime risk of developing PC, while Central Africa has the lowest.

According to Cancer Statistics 2024, an estimated 66,440 new cases of PC will be diagnosed in the U.S. in 2024, with 34,530 cases in men and 31,910 in women. In addition, there will be 51,750 deaths from PC, with 27,270 deaths in men and 24,480 in women [27]. Although PC accounted for only 3.2% of all new cancer diagnoses in 2022, it was responsible for 8.2% of all cancer-related deaths, with the majority being pancreatic ductal adenocarcinoma (PDC) [2,28]. Due to its poor survival rates, the age distribution of PC deaths closely mirrors that of diagnoses, with an ASR of 11.2 per 100,000 for both men and women annually, based on data from 2018–2022 [2].

Since long-term survival for patients diagnosed with PC largely depends on small, resectable tumor size and early-stage diagnosis, strategies for improving early detection and screening should be prioritized [29]. However, given its relatively low incidence (2.6%) compared to lung cancer (12.4%), female breast cancer (11.6%), and colorectal cancer (9.6%), with a lifetime risk of 0.64% in men and 0.44% in women, screening is not currently recommended for the general population [1,30]. For individuals at high risk due to genetic predispositions or family history, the American Gastroenterological Association (AGA) and the American Society for Gastrointestinal Endoscopy (ASGE) both recommend endoscopic ultrasound (EUS) and magnetic resonance imaging (MRI) as the preferred screening modalities [31,32].

## 3. Changes in Incidence

**Figure 1:**
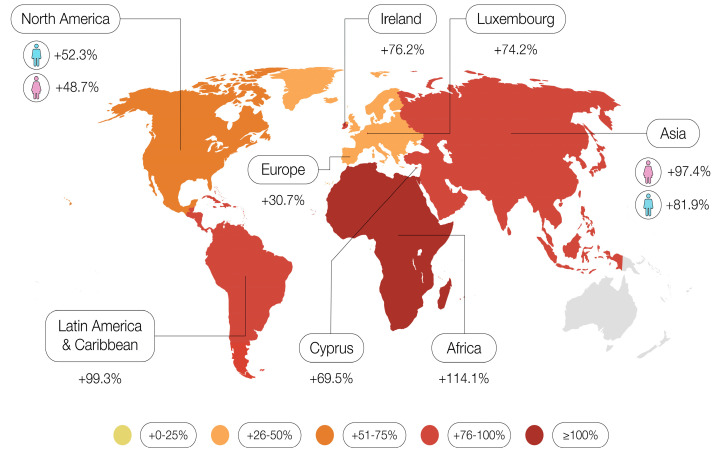
Global trends in pancreatic cancer incidence from 1990–2021 as presented in the GBD study 2021.

According to data extracted from the GBD Study 2021, the global incidence rate of PC increased by 8.9%, rising from 5.47 per 100,000 in 1990 to 5.96 per 100,000 in 2021 [33]. When comparing countries by SDI, which includes variables related to global economic conditions, educational levels, and fertility rates [34], it was observed that between 1990 and 2021, the low-middle SDI region experienced the largest increase in PC incidence rates (0.58). By 2021, the high-SDI region had the highest incidence rate at 10.00 per 100,000 (95% UI, 9.08–10.61), while the low-SDI region reported the lowest rate at 1.59 per 100,000 (95% UI, 1.33–1.90) [35] (Figure 1).

A study by Huang et al., analyzing recent trends in PC across 184 countries, including registries from 48 countries through 2017, revealed significant increases in incidence among men in 14 countries (AAPCs: 8.85–0.41) and among women in 17 countries (6.04–0.87) [25]. The highest incidence rates for men were observed in Iceland (AAPC 8.85), Cyprus (5.51), and France (4.30), while Costa Rica was the only country with a decreasing trend (−4.15). Among women, several countries showed rising incidence, notably Malta (AAPC 6.04), Slovakia (4.40), and France (4.22), while Denmark was the only country with a decline (−2.43).

When examining incidence rates by stage, the rates per 100,000 people in 2004 were 1.0 for localized stages, 3.0 for stages with regional metastases, and 6.0 for stages with distant metastases. In comparison, the rates in 2021 were 2.4 for localized stages, 3.5 for regional metastasis, and 6.5 for distant metastases [2].

A cohort study published by Koh et al. in 2023, analyzing incidence rates of early-onset cancers in the U.S. between 2010 and 2019, showed that gastrointestinal cancers had the fastest-growing incidence rates among all organ systems, with an ASIR of 11.49 per 100,000 in 2010 compared to 13.65 per 100,000 in 2019, reflecting an AAPC of 2.16% [36]. PC showed an AAPC of 2.53%, with an ASIR of 1.06 per 100,000 in 2010, rising to 1.3 per 100,000 in 2019.

Traditionally considered a disease of the elderly, with a median diagnosis age of 70 years [2]. PC now shows a rising incidence among younger individuals, according to recent data [37].

According to Cancer Research UK, the ASR for PC in the UK increased by 12% between 2003–2005 and 2017–2019. Comparing ASR across all age groups and sexes since the early 1990s, the rates have increased by 308% in individuals aged 0–24, 42% in 25–49-year-olds, 23% in 50–59-year-olds, 12% in 60–69-year-olds, 21% in 70–79-year-olds, and 19% in those aged 80 and older for females. Among men, the rates showed increases of 14% in those aged 60–69, 18% in those aged 70–79, and 15% in those aged 80 and older [38].

A study by Gaddam et al. in 2021 analyzed the incidence rates of PC in U.S. adults from 2000 to 2008, revealing that AAPCs increased by 0.62% in women aged 55 and older, with higher rates in men aged 55 and older (0.92%) [39]. However, when evaluating the incidence trends among individuals younger than 55, women showed a significantly higher increase in incidence (1.93%) compared to men (0.77%).

A population-based time-trend analysis by Abboud et al. (2024), using data from the National Program of Cancer Registries (NPCR), showed an increasing age-specific incidence of PC, particularly among younger women [40]. The analysis of nationwide data from the U.S. between 2001 and 2018 revealed a significant increase in the ASIR for women (AAPC: 1.11%, 0.84–1.37%) and men (AAPC: 1.17%, 1.05–1.30%) for individuals aged ≥55 years. However, when comparing patients younger than 55 years, the ASIR increased at a faster rate in younger women (AAPC: 2.36%, 1.97–2.75%) compared to men (AAPC: 0.62%, 0.03–1.22%).

A systematic analysis of the GBD study by Dahia et al. (2024) found that global incidence, mortality, and disability-adjusted life years (DALYs) for EOPC rose significantly from 0.64, 0.56, and 26.67 per 100,000 in 1990 to 0.94, 0.81, and 37.85 in 2021, with percentage changes of +0.3, +0.25, and +11.18, respectively [41]. The incidence, death, and DALY rates for males showed a greater percentage increase compared to those for females, with changes of 0.36, 0.32, and 14.21 for males and 0.22, 0.18, and 8.11 for females.

In a cross-sectional study by Li et al. (2024), based on the GBD 2019 data, the ASIR for early-onset PC in individuals younger than 50 years increased by 46.9%, with an AAPC of 1.26 [42]. When considering sex differences, men (ASIR 1.19) had an incidence rate approximately 1.8 times higher than the global average, while women had an ASIR of 0.67 [43,44].

However, a recent analysis published by Patel et al. (2024) suggested that the rise in PC incidence may be due to improved detection of early-stage endocrine cancers rather than an actual increase in pancreatic adenocarcinoma (PAC) [45]. This reflected previously undiagnosed cases. Despite the increased incidence of PC in young adults aged 15–39 from 2001 to 2019 (AAPC: 4.8% for women, 2.7% for men), as well as an increase in early-stage cancer (AAPC: 11.7% for women and 11.1% for men), the rise was attributed to endocrine cancers and solid pseudopapillary neoplasms. The study showed an AAPC of 7.3% for endocrine cancers in women and 7.5% in men, and an AAPC of 14.4% for pseudopapillary neoplasms. In line with these observations, the incidence of small tumors (≤2 cm) increased significantly, rising about eightfold in women (from approximately 0.22 to 1.8 per million) and tripling in men (from around 0.33 to 1 per million).

Analyzing PC incidence trends, the data indicate a significant overall increase in incidence (+77%) between 2018 and 2040. Africa experienced the highest increase (+114.1%), followed by Latin America and the Caribbean (+99.3%). In North America, men are expected to have higher PC incidence rates (+52.3% in men vs. +48.7% in women), and similarly in Europe (+30.7% in men vs. +27.8% in women). However, in Asia, Latin America and the Caribbean, and Oceania, women are projected to have higher incidence rates. In Africa, the rates are expected to be equal for both sexes [46].

Looking at predictions for the European Union’s (EU) PC burden, the number of new cases is expected to increase significantly by 2040. The highest projected increases in incidence are in Ireland (+76.2%), Luxembourg (+74.2%), Cyprus (+69.5%), Spain (+45.9%), the Netherlands (+44.4%) and the United Kingdom (UK) (+41.4%). In countries where the population’s age is expected to remain relatively stable until 2040, the number of new cases is expected to remain unchanged or increase at a slower pace. These trends are seen in Bulgaria (−0.2%), Latvia (+4.3%), Lithuania (+8.5%), and Hungary (+14.9%) [47].

Regarding U.S. PC projections, it is estimated that by 2030, there will be 40,000 new cases in men and 36,000 new cases in women. By 2040, these numbers are expected to rise to 48,000 new cases in men and 45,000 new cases in women [22].

## 4. Changes in Mortality

According to Cancer Statistics 2024 published by the American Cancer Society, PC is the third leading cause of cancer-related death in the U.S. in both men and women combined. Reflecting incidence patterns, the mortality rate from PC has increased by 0.3% annually since 2000 [27].

A systematic analysis of the GBD study 2021 examined mortality, incidence, and disability-adjusted life years (DALYs) for PC from 1990 to 2021. The analysis revealed a significant global rise in PC mortality over the study period. Deaths from PC increased from 211,613 in 1990 to 505,752 in 2021. The ASMR rose from 5.655 to 5.948 per 100,000 population, with an estimated annual percentage change (EAPC) of 0.208%. Notably, low–middle SDI regions saw a marked increase (EAPC: 1.553%), with deaths rising from 8671 to 32,553. The highest ASMRs in 2021 were reported in Central Europe (9.275) and high-income Asia Pacific (9.563), compared to the lowest in South Asia (1.512) and Western Sub-Saharan Africa (2.091). Significant regional trends were observed, with Western Sub-Saharan Africa, Central Asia, and Southeast Asia experiencing EAPCs of 2.393%, 1.367%, and 1.274%, respectively [22,33]. The low–middle SDI region showed the most dramatic rise in mortality rates (+0.57), while the high-SDI region recorded the highest rate in 2021 at 9.38 per 100,000. In contrast, the low-SDI region had the lowest rate at 1.73. Males consistently had higher mortality than females across all regions, and individuals aged 70 and above experienced the highest global mortality rate at 56.91 per 100,000 [33].

**Figure 2:**
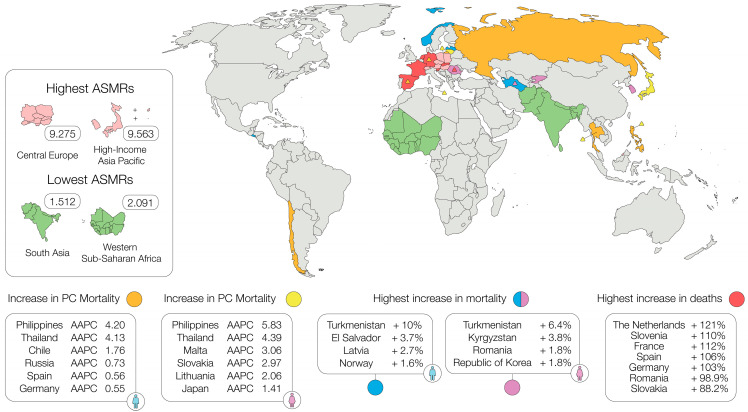
Global trends of PC mortality from 1990–2020.

Looking at mortality trends for PC over the last three decades, data published by Ilic et al. (2022) showed significant increases in mortality in both sexes across most countries [18]. The highest average AAPCs for men were seen in Turkmenistan (+10%), El Salvador (+3.7%), Latvia (+2.7%), and Norway (+1.6%). Similarly, for women, the highest increases were observed in Turkmenistan (+6.4%), Kyrgyzstan (+3.8%), Romania, and the Republic of Korea (both +1.8%) (Figure 2).

Using GLOBOCAN database data, Huang et al. showed a marked increase in PC mortality in eight countries among men, with significant increases in Russia (AAPC: 0.73), Spain (AAPC: 0.56), and Germany (AAPC: 0.55) in Europe, and in the Philippines (AAPC: 4.20), Thailand (AAPC: 4.13), and Chile (AAPC: 1.76) in other regions. Among women, 14 countries showed an increasing trend in PC mortality (AAPCs ranging from 5.83 to 0.78), with the sharpest rises in Malta (AAPC: 3.06), Slovakia (AAPC: 2.97), and Lithuania (AAPC: 2.06) in Europe, and in the Philippines (AAPC: 5.83), Thailand (AAPC: 4.39), and Japan (AAPC: 1.41) in other regions [25] (Figure 2).

In the U.S., a CDC population-based study published in 2024 analyzed PC mortality from 1999 to 2020. The study found that PC caused 810,628 deaths in the U.S. over this period, with an average of nearly 39,000 deaths annually. The ASMR rose slightly from 10.6 to 11.1 per 100,000 population, with similar increases for men (from 12.3 to 12.7) and women (from 9.3 to 9.6). Men had a consistently higher average ASMR (12.5) compared to women (9.5) [48].

European data from the GBD study (1990–2019) published in the *International Journal of Cancer* (2021) showed a substantial increase in the incidence, mortality, and DALYs for PC in the EU (EU-28, pre-Brexit). Deaths from PC increased from 59,500 in 1990 to 108,800 in 2019, with an EAPC of 2.23%. The ASMR was 8.6 in 1990, compared to 9.9 in 2019, reflecting an EAPC of 0.55%. The countries with the highest increase in death cases were The Netherlands (+121%), Slovenia (+110%), France (+112%), Spain (+106%), Germany (+103%), Romania (+98.9%), and Slovakia (+88.2%). Interestingly, in 1990, PC cancer mortality slightly exceeded incidence; however, by 2019, the opposite was true, suggesting that underreporting of cases in 1990 may have contributed to this discrepancy [49]. This trend aligns with research suggesting that the rise in PC mortality may be attributed to improvements in diagnosis and certification in certain regions [50,51,52] (Figure 2).

According to the WHO, deaths from PC are projected to increase significantly in the coming decades, reaching approximately 300,000 deaths in 2030, 400,000 in 2040, and 485,000 in 2050 for men. For women, the projections are 280,000 in 2030, 365,000 in 2040, and 450,000 in 2050. A cross-continental analysis of projections for 2040 indicates the following increases in PC-related deaths:

- Africa: +82.9% for men, +81.9% for women.
- Asia: +58.2% for men, +62.4% for women.
- Latin America and the Caribbean: +60.8% for men, +56.7% for women.
- Northern America: +52.3% for men, +44% for women.
- Oceania: +65.9% for men, +63.5% for women.
- Europe: +30.1% for men, +21.4% for women.

This increase will be more pronounced in low-HDI (+77.1% males, +82.6% females) and medium-HDI countries (+67.1% males, +66.7% females) compared to high-HDI (+57.5% males, +64.0% females) and very-high-HDI countries (+41.6% males, +33% females) [53].

For the EU in 2024, the projected ASMR is 8.21, which represents a 1.59% increase compared to the observed rates in 2018 [54].

## 5. Changes in Survival

According to the Cancer Facts and Figures report published by the American Cancer Society in 2024, the 5-year relative survival rate for all stages of PC combined is 13%. This varies significantly between types of PC, with exocrine tumors showing a 5-year survival rate of 8%, compared to neuroendocrine tumors (NETs), which have a much higher survival rate of 72%. Survival rates based on stage at diagnosis (2013–2019) show the following: 44% for patients diagnosed with localized disease, 16% for those with regional disease, and only 3% for patients diagnosed with distant metastases [55]. By comparison, the 5-year relative survival rate for PC was as low as 3% between 1975 and 1977 and 4% between 1995 and 1997 [27].

One of the largest studies evaluating the long-term survival of PDAC in the modern era, published by Bengtsson et al. (2020), utilized population-level data from the SEER database [56]. This study included patients diagnosed with microscopically confirmed PDAC from 1975 to 2011. The data revealed that the 5-year survival rate for all patients with PC rose significantly from 0.9% to 4.2% over this period. Notably, patients who underwent surgical resection saw a substantial improvement, with survival rates rising from 1.5% to 17.4%. In contrast, the survival rates for non-resected patients showed minimal change, increasing slightly from 0.8% to 0.9%. For surgically resected patients diagnosed between 2004 and 2011, factors such as age, gender, tumor grade, size, TNM stage, and chemotherapy were significant predictors of survival. In non-resected cases, age, tumor grade, and TNM stage were the primary survival predictors.

Another review published in 2022, analyzing data from nine registries of the National Cancer Institute between 1975 and 2016, found that 5-year survival rates for PC in the U.S. modestly improved, rising from 2% in the late 1970s to 9.2% by 2011. Early detection and tumor stage at diagnosis were identified as key factors for these improved outcomes [57]. Additionally, an analysis by Chen et al. (2024) indicated a significant increase in survival rates from 6.3% between 2002 and 2006 to 12.4% between 2015 and 2019 [58]. Another study by Li et al. (2020) showed that the relative survival rate (RSR) for patients diagnosed with PC increased from 7.9% in 2002 to 23.7% in 2016, with predictions suggesting a further increase to 33.9% for patients diagnosed between 2017 and 2021 [59].

In Europe, data from the EUROCARE-5 Working Group on cancer survival (1999–2007) showed that the highest 5-year survival rates for PC were observed in Croatia (10.9%) and Belgium (10.5%) for both sexes, while Malta (0%) and Northern Ireland (3.02%) reported the lowest rates [46,60]. According to Cancer Research UK, the one-year age-standardized net survival rate for PC rose from 10% in 1971–1972 to 22% in 2010–2011 for men, an increase of 11 percentage points. For women, the survival rate improved from 11% to 20% over the same period, a 9-percentage-point increase. However, there was no significant improvement in the 5-year and 10-year survival rates for PC [38].

A study showing long-term survival trends for PC in Nordic countries found increases in the 1-year survival rates from 1970–1979 to 2010–2019 in both men and women. For instance, in Denmark, the 1-year survival rate rose from 9.1% to 32.1% for men and from 10% to 35.3% for women. In Finland, the rate improved from 11.2% to 28.4% for men and from 12.7% to 33.3% for women. Norway showed increases from 11.3% to 36.1% for men and from 13.2% to 37.3% for women, while in Sweden, the rates increased from 11.2% to 37.1% for men and from 11.5% to 38.9% for women [61].

The 5-year survival rates also showed gradual improvement, rising from about 2% in all countries and sexes to 9.3% for men and 10.5% for women in Denmark, 7.5% for men and 8.9% for women in Finland, 12.8% for men and 13.4% for women in Norway, and 11.8% for men and 12.8% for women in Sweden. The gap between the 1-year and 5-year survival rates grew over time, exceeding 20 percentage points in the most recent period, likely reflecting earlier diagnosis and improved, though non-curative, treatments [61].

## 6. Reasons for Increasing Numbers

A study by Huang et al. (2024) on PC incidence and mortality across 184 countries, examining associations with lifestyle and metabolic risk factors, found that higher ASR of PC incidence and mortality in men were significantly linked to an increased prevalence of smoking, alcohol consumption, physical inactivity, obesity, hypertension, and high cholesterol [25]. Similar trends were observed in women, particularly regarding smoking, alcohol consumption, and high cholesterol.

In a study by Islami et al. (2024), which estimated the proportion and number of invasive cancer cases and deaths in the U.S. in 2019 attributable to modifiable risk factors (including cigarette smoking, secondhand smoke, excess body weight, alcohol consumption, and red/processed meat consumption), the population-attributable fraction (PAF) was found to be 13.9% for both sexes (15.5% in men, 12.1% in women) for cigarette smoking and 17.9% (17.4% in men, 18.5% in women) for excess body weight in PC [62].

A 2025 global analysis for the GBD 2021 highlighted several risk factors contributing to PC mortality. It reported an increase in the contribution of high body mass index (BMI) (from 3.19% in 2000 to 4.69% in 2021), high fasting plasma glucose (from 27.24% to 35.78%), and smoking (from 16.45% to 13.84%) to all-age deaths due to PC. The ASMR per 100,000 for these risk factors in 2021 was 0.45 (up from 0.31 in 2000) for BMI, 3.33 (up from 2.48 in 2000) for high plasma glucose, and 1.34 (down from 1.57 in 2000) for smoking [63].

The GBD study further notes that the global rise in PC cases and deaths, particularly in low- and middle-SDI countries, is largely driven by aging populations, environmental and behavioral changes, and increased exposure to risk factors, rather than genetic factors. From 1990 to 2017, ASIR and ASMR increased globally, with the highest rates observed in high-SDI regions, likely due to aging populations and lifestyle-related risk factors like obesity and diabetes. Despite a slight reduction in smoking rates in high-income countries, the overall global burden of PC has more than doubled, driven by population growth and an increase in risk factor prevalence [64].

According to GBD data, the contributions to PC deaths were as follows: high fasting plasma glucose accounted for 26%, tobacco use for 14%, and high BMI for 2%. In high-SDI regions, the fraction for high fasting plasma glucose was 28%, compared to 23% in low-SDI regions. Over the past 30 years, deaths attributable to high fasting plasma glucose increased from 19% to 26%, while deaths due to high BMI rose from 0.4% to 2%, and those linked to tobacco use declined from 17% to 14%. In 2021, North Africa, the Middle East, and high-income North America reported the highest proportions of deaths due to high BMI (4.7% and 4.6%, respectively), while high-income North America had the highest impact from fasting plasma glucose (34%). Tobacco-related deaths were most prevalent in East Asia (19%) and lowest in Western Sub-Saharan Africa (4%) [33].

### 6.1. Demographic Shifts

Demographic and epidemiologic shifts are occurring worldwide, particularly over the last century [65,66]. With advancements in public health, life expectancy has increased in many countries, while urbanization has contributed to a decline in birth rates [67,68,69]. Since PC predominantly affects older individuals—90% of new cases occur in people over the age of 55, with the most common diagnoses in individuals in their 60s and 70s [70,71]—age is one of the most significant risk factors for its development [43,72]. Data show that the global population aged 65 and older grew from 6.1% to 8.8% between 1990 and 2017, accompanied by an increase of 9 million deaths worldwide during the same period [73]. The WHO predicts that the number of elderly individuals diagnosed with PDAC will rise with increasing life expectancy, with 61% to 70% of all cancers, including PDAC, expected to be diagnosed in adults over 65 by 2030 [74,75].

Analysis from the GBD study (2021) found that the highest number of incident cases occurred in the 70–74 age group, with a total of 81,736 cases. The highest incidence rate across all age groups was recorded in the 90–94 age group, with 103.59 per 100,000 population, accounting for 20.5% of the global PC incidence in 2021 [35].

### 6.2. Tobacco Smoking and Chronic Pancreatitis

Tobacco smoking is one of the strongest modifiable risk factors for the development of PC [76]. Studies have shown that smokers not only face a significantly higher risk of developing PC compared to non-smokers [77] but also experience earlier onset of the disease [17,78]. The relative risk (RR) of developing PC is 1.8 for current smokers and 1.2 for former smokers. For those smoking more than 30 cigarettes a day, the relative risk increases to 2.2 [79,80]. Over the past 30 years, smoking-related deaths have declined, largely due to increased tobacco control measures and reduced consumption [81,82]. According to the GBD study, most of the deaths and disability-adjusted life years (DALYs) attributable to tobacco smoking are concentrated in Central and East Asia, particularly among individuals aged 40–60 years [33].

Furthermore, tobacco smoking has been shown to play a dose-dependent role in the development of chronic pancreatitis, contributing as a primary cause in up to 25% of cases [83,84]. However, alcohol consumption is the leading risk factor for chronic pancreatitis, responsible for 44–65% of cases [85,86,87]. While heavy alcohol consumption (around 80 g/day for six years) is considered the threshold for developing chronic pancreatitis, even lower levels of alcohol intake can contribute to disease progression when combined with other risk factors [88,89]. Chronic pancreatitis itself is a significant risk factor for PC, with studies reporting PC incidence in chronic pancreatitis patients ranging from 0.68% to 2.94%, depending on patient selection and study methodology [90,91,92,93,94].

A 2024 study by Li et al., analyzing GBD 2021 data, showed a 12.8% reduction in ASI for chronic pancreatitis between 1990 and 2021 [42]. However, urbanization and rising affluence in developing nations, particularly in China and India, have led to increased alcohol consumption and a corresponding rise in chronic pancreatitis cases [95,96].

### 6.3. Impact of the COVID-19 Pandemic

The WHO declared COVID-19 a global pandemic in March 2020 [97], prompting widespread restructuring of healthcare systems to prioritize critically ill patients [98]; as a result, oncology services were heavily disrupted [99,100], with delays in cancer screening, diagnosis, and treatment, particularly affecting high-risk patients such as those with severe comorbidities or active malignancies [101,102,103]. The CAPANCOVID study (2022), a French comprehensive multicenter observational cohort study, suggests the presence of missed diagnoses and a shift in disease stage at diagnosis from resectable to advanced disease, accompanied by changes in treatment strategy [104]. During the first lockdown, weekly number of newly diagnosed pancreatic cancer cases dropped by 18.2 (from 13.2 to 10.8 cases), with no rebound observed in the post-lockdown period (13.2 to 12.9 cases). The proportion of borderline tumors significantly increased from 13.6% to 21.7%, while metastatic cases significantly decreased from 47.1% to 40.3%. After the lockdown, borderline tumors significantly declined to 9.6%, and the proportion of advanced-stage disease significantly rose to 69.8%.

Similarly, a study published by Salirosas et al. (2024) showing changes in the number of diagnoses and treatment patterns for PC patients during the pandemic indicated that, among 127,613 patients with PDAC, the number of new diagnoses rose from 30,573 in 2017 to 33,465 in 2019, followed by a decline to 31,218 cases in 2020 [105]. Furthermore, the study showed that between 2017 and 2019, the proportion of patients receiving surgery or radiotherapy remained stable, but both declined in 2020, while the previously rising use of chemotherapy plateaued. Additionally, the average time from diagnosis to surgery increased significantly—from 34 days in 2017 to 81 days in 2020—and was even longer in patients tested for COVID-19, regardless of test result (140 days for positive, 112 days for negative).

The UK CONTACT study (2023), a national observational cohort study, also showed that fewer patients received standard treatments [106]. In a cohort of 984 patients (pre-COVID-19: 483; COVID-19: 501), those diagnosed during the COVID-19 period were less likely to undergo advanced staging (29.5% vs. 37.2%), more often received upfront chemotherapy instead of surgery (45.5% vs. 23.4%), and ultimately underwent fewer surgical resections (6.4% vs. 9.3%), while a greater proportion received no anti-cancer treatment at all (69.3% vs. 59.2%). Interestingly, despite differences in treatment approaches during the pandemic, there was no significant difference in median overall survival between the COVID-19 and pre-COVID-19 cohorts (3.5 vs. 4.4 months). Furthermore, a retrospective multicenter study from Paris reported a 29% decline in pancreatic cancer referrals during the first COVID-19 lockdown yet found no significant stage shift or change in treatment allocation, and one-year overall survival remained comparable between 2019 and 2020–2021 across the treatment groups: 92% vs. 89% for surgery, 52% vs. 56% for systemic therapy, and 13% vs. 10% for best supportive care [107]. In contrast, a study by Madge et al. reported a significant reduction in survival among pancreatic ductal adenocarcinoma patients diagnosed during the pandemic, with median survival nearly halved compared to the pre-pandemic cohort (3.3 vs. 7.4 months), and the greatest decreases observed in disease stages 2 and 3 (–9.8 and –3.8 months, respectively); this difference persisted even after excluding COVID-19-related deaths [108]. The studies show varying impacts on survival, suggesting that the long-term prognostic effects of pandemic-related disruptions remain uncertain.

### 6.4. Diabetes as a Major Risk Factor

The link between metabolism and cancer is well established, with metabolic dysfunction being a key characteristic of cancer, and obesity and diabetes representing the most common metabolic risk factors [109,110]. Around 85% of PC patients are diagnosed with concurrent diabetes, and those with type 2 diabetes for five or more years have a 50% higher risk of developing PC compared to non-diabetic individuals [109]. A prospective study from the China Kadoorie Biobank found that individuals with previously diagnosed or screen-detected diabetes had a twofold increase in the risk of developing PC (Hazard ratio (HR) = 1.87) [90]. A meta-analysis of 22 studies also showed that for every 1 mmol/L increase in blood glucose, individuals without diabetes had a 15% increased risk of PC (HR = 1.15) [111].

The Fremantle Diabetes Study found that the incidence of PC was twice as high in adults with type 2 diabetes compared to those without it over a follow-up period of up to 25 years (Incidence Rate Ratio (IRR) = 2.06) [112]. Additionally, a prospective cohort study by Xia et al. (2020) showed that participants with metabolic syndrome (HR = 1.31), central obesity (HR = 1.24), and hyperglycemia (HR = 1.60) had an increased risk of developing PC [113]. A nationwide study of 1.79 million Israeli adolescents with a median follow-up of 23 years found that obesity was a higher cancer risk factor for both men (HR 3.67) and women (HR 4.07). The study estimated the population-attributable fraction due to overweight and obesity at 10.9% [114].

A pooled analysis involving 141 million participants published in 2024 examined trends in diabetes prevalence from 1990 to 2022. The study found that the age-standardized prevalence of diabetes increased in 131 countries for women and 155 countries for men, with the most significant rises observed in low- and middle-income countries in regions such as Southeast Asia, South Asia, the Middle East, North Africa, and Latin America. In contrast, prevalence remained stable in parts of Europe, sub-Saharan Africa, East Asia, and Canada, while it declined in women in Japan, Spain, and France, and in men in Nauru. Additionally, by 2022, it was estimated that 445 million adults aged 30 or older were living with untreated diabetes, accounting for 59% of all diabetes cases, a 3.5-fold increase from 1990. Although treatment coverage has improved in many countries, especially in Europe, Latin America, Canada, and South Korea, coverage remains low in sub-Saharan Africa, South Asia, and some Pacific nations, with some African countries reporting rates below 10% [114].

According to the Diabetes Atlas published in 2022 by The International Diabetes Federation (IDF), the global diabetes prevalence among adults aged 20–79 was estimated at 10.5% (536.6 million people) and is projected to rise to 12.2% (783.2 million) by 2045. Data indicate prevalence to be higher in urban (12.1%) than rural (8.3%) areas, as well as in high-income (11.1%) compared to low-income countries (5.5%), with the highest relative increase in diabetes cases by 2045 expected in middle-income countries (21.1%), followed by high- (12.2%) and low-income (11.9%) countries [115].

A systematic analysis for the Global Burden of Disease Study 2021 published in 2023 showed that between 1990 and 2021, the global age-standardized prevalence of diabetes nearly doubled, increasing from 3.2% to 6.1%. Increases of more than 100% were seen in North Africa and the Middle East (161.5%) and the high-income super-region (114.8%). Considering the increase in diabetes prevalence, six regions (North Africa and the Middle East, high-income North America, central Asia, Oceania, Andean Latin America, and southern Latin America) showed increases of 100% and an additional six regions (Western Europe, southern sub-Saharan Africa, eastern Europe, south Asia, high-income Asia Pacific, and central sub-Saharan Africa) showed an increase of more than 90%. By 2050, an estimated 1.31 billion (1.22–1.39) people will have diabetes, with age-standardized prevalence exceeding 10% in North Africa and the Middle East (16.8%; 16.1–17.6) and Latin America and the Caribbean (11.3%; 10.8–11.9), while 89 out of 204 countries (43.6%) are projected to surpass this threshold [116]. Considering the U.S. population, showing significant increasing cases of pancreatic cancer in both men and women [39], a clear upward trend over time can be seen, with age-standardized prevalence rising significantly from 9.8% in 1999–2000 to 14.3% in 2017–2018 [117].

### 6.5. Obesity on the Rise

Over 50% of global type 2 diabetes DALYs are attributed to high BMI [42]. Obesity is a significant risk factor for both diabetes and PC. A 2024 pooled analysis of 3663 population-representative studies showed that from 1990 to 2022, the age-standardized prevalence (AGP) of obesity increased in 188 countries for women and almost all countries for men, with the most significant rises seen in sub-Saharan Africa (women), the U.S., Brunei, Central Europe, and Polynesia/Micronesia (men), as well as in the Caribbean and the Middle East/North Africa. In 49 countries, obesity prevalence among women rose by more than 20 percentage points, and 24 countries showed similar increases among men. The largest increases were recorded in The Bahamas for women (33.0 percentage points) and Romania for men (31.7 percentage points) [118].

In the U.S., obesity is a significant health burden, with age-standardized prevalence rates (ASPR) increasing by 158.4% among male adolescents and 185.9% among female adolescents from 1990 to 2021. Among adults, obesity prevalence rose by 123.6% in males and 99.9% in females. Projections suggest that by 2050, an additional 3.41 million adolescents and 41.4 million adults will have overweight or obesity, bringing the total number of affected adults to 213 million [119].

### 6.6. Increasing Incidence of PC in Younger Individuals

There has been a notable increase in the incidence of PC among younger individuals (ages 20–49) over recent decades [39,40,41,44]. However, the etiological factors driving this rise remain unclear. A systematic literature review by Sreenivisa et al. identified several potential non-heritable risk factors for EOPC, including smoking, alcohol consumption, pancreatitis, and hepatitis B infection, but these require further investigation [120].

In 2024, data from Dahia et al. revealed that, between 1990 and 2019, the highest proportion of disability-adjusted life years (DALYs) due to EOPC in Eastern Europe was attributed to smoking and tobacco use, especially among males (0.21) and overall (0.24) [41]. For females, the highest proportion was observed in Western Europe (0.24). High BMI was the leading contributor to DALYs in high-income North America, accounting for 0.21 overall, 0.15 in females, and 0.18 in males. High fasting plasma glucose contributed most to DALYs in Oceania, with proportions of 0.07 overall, 0.11 in females, and 0.09 in males. However, across SDI regions, smoking and tobacco use contributed the most in high-middle SDI regions for males (0.12) and high-SDI regions for females (0.18) and both sexes combined (0.17).

### 6.7. Tobacco Smoking and Its Continuing Impact Among Younger People

Although the prevalence of tobacco smoking has decreased significantly among young people (with a reduction of 32.9% in males and 37.6% in females between 1990 and 2019), smoking prevalence in 2019 still exceeded 20% among males in 120 countries and among females in 43 countries [121]. A study evaluating genetic and non-genetic risk factors for EOPC, involving 3280 patients, showed that current smoking has a stronger impact on the risk of developing PDAC in individuals under 50 years of age (odds ratio (OR) = 2.92) compared to older individuals (OR = 1.51) [122].

### 6.8. Global Trends in Young-Onset PC

In a 2024 study by Cai et al., which analyzed global trends of PC in young adults using the GBD dataset, a notable increase in incidence was observed, particularly in regions such as South America, North America, Oceania, and Africa [123]. The study found a negative correlation between the Human Development Index (HDI) and the incidence of EOPC, with a positive association between HDI growth from 1990 to 2019. The U.S. showed a significant rise in EOPC cases, likely driven by the high and worsening prevalence of obesity and diabetes, even among younger individuals. Additionally, PC mortality linked to high BMI increased from 0.32 to 0.49 cases per 100,000 people between 1990 and 2019, with the increase more pronounced among young adults from 2012 to 2019. This period saw a growth rate of 7.6 × 10^−8^ per year, more than double that of the 1990–2011 period (3.6 × 10^−8^ per year).

### 6.9. Survival Outcomes in Early-Onset vs. Later-Onset PC

EOPC is associated with worse survival outcomes compared to LOPC. A study by Ansari et al. comparing outcomes between patients with EOPC and LOPC showed that EOPC was linked to significantly lower 5-year OS (6.1% vs. 8.6%) and cancer-specific survival (CSS) (6.7% vs. 9.7%) [124]. EOPC remained a significant risk factor for poorer OS and CSS, with lower survival rates observed in operated patients (5-year OS: 17.7% vs. 26.9% and 5-year CSS: 18.9% vs. 29.7%).

## 7. Reasons for Improved Survival

### 7.1. Advancements in Surgery and Centralization

Surgical resection (pancreatectomy) remains the only established curative approach for patients with PDAC [125], with multiple studies confirming that surgery is linked to improved survival [126,127,128]. Advances in surgical techniques, including artery-first approaches, radical antegrade modular pancreatosplenectomy, and refined methods for venous and arterial resection, have improved the completeness of PDAC resections and expanded surgical options, even in cases with vascular involvement [129].

Due to this advancement, mortality rates after surgery have significantly improved. Data from the American College of Surgeons National Surgical Quality Improvement Program (ACS-NSQIP) showed that among 28,888 patients, postoperative mortality after pancreaticoduodenectomy significantly decreased by 4.58% annually despite a slight rise in major morbidity, whereas for distal pancreatectomy, no significant changes in morbidity or mortality rates were observed over time [130]. Furthermore, an analysis published by Del Valle et al. (2021) using ACS-NSQIP for 32,165 patients who underwent pancreaticoduodenectomy from 2006 to 2016 showed postoperative mortality significantly decreased from 2.9% to 1.5% (OR 2.55), while the incidence of major morbidity remained unchanged (26.8% to 25.9%, OR 1.22) and failure to rescue improved markedly, dropping from 9.8% to 4.1% (OR 3.65) [131].

A multicenter prospective snapshot study in 67 countries published in 2024 indicated that the overall 90-day postoperative mortality rate was 5.4% (229 of 4223 patients), with a significantly higher risk observed in the low-to-middle HDI group (adjusted OR 2.88) [132].

Furthermore, evidence emerging in the late 1990s and early 2000s demonstrated that treatment at high-volume surgery centers and Centralization of Surgical Care and Multimodality Therapy for Pancreatic Adenocarcinoma significantly improved perioperative outcomes, reduced short-term mortality, and enhanced long-term survival, a finding consistently supported by more recent data [133,134]. A retrospective cohort study by Hoehn et al. (2024) showed that in a cohort of 4141 patients, multidisciplinary clinic (MDC) management improved access to neoadjuvant chemotherapy (OR 3.33), surgery (OR 1.39), and clinical trials (OR 3.76), eliminating the survival disadvantage typically seen in low-socioeconomic-status patients treated outside the MDC [135].

A large German cohort study (2022) involving over 45,000 pancreatic cancer patients found that treatment in certified cancer centers significantly improved survival, with median survival increasing to 8.0 months compared to 4.4 months in non-certified hospitals, and an 11% reduction in long-term mortality [136]. Another retrospective study by Hsu et al. (2022), including 1621 pancreatic cancer patients, demonstrated that centralization of care within an integrated healthcare system improved median overall survival by 3 months and significantly increased the use of neoadjuvant chemotherapy from 10% to 31% [137].

### 7.2. Perioperative Chemotherapy

The use of perioperative systemic therapy has enabled the downstaging of tumors, thereby rendering previously unresectable lesions operable, leading to higher rates of R0 resections and improved overall survival outcomes [138,139].

The Phase II NUPAT-01 study (2022) evaluated the use of neoadjuvant chemotherapy with FOLFIRINOX or gemcitabine/nab-paclitaxel in patients with borderline resectable pancreatic cancer, demonstrating promising outcomes in terms of resection rates and survival. A total of 84.3% of patients underwent surgery following neoadjuvant chemotherapy with FOLFIRINOX or GEM/nab-PTX, achieving an R0 resection rate of 67.4%, a 3-year overall survival rate of 54.7%, and a median survival time of 39.4 months, with the treatment proving feasible, well tolerated, and without significant survival differences between the regimens.

In a prospective, randomized Phase 2/3 trial published by Jang et al. (2018) neoadjuvant chemoradiation significantly improved outcomes in patients with borderline resectable pancreatic cancer, achieving a higher 2-year survival rate (40.7% vs. 26.1%), longer median survival (21 vs. 12 months), and a markedly increased R0 resection rate (51.8% vs. 26.1%) compared to upfront surgery, leading to early study termination due to clear efficacy [140]. The Dutch Randomized PREOPANC Trial (2022) also showed that neoadjuvant chemoradiotherapy significantly improved overall survival compared to upfront surgery (HR 0.73), leading to a notably higher 5-year survival rate (20.5% vs. 6.5%) despite only a modest difference in median survival (15.7 vs. 14.3 months), with consistent benefits across both resectable and borderline resectable pancreatic cancer subgroups [141].

### 7.3. Advancements in Chemotherapy Regimes

The introduction of multi-agent chemotherapy regimens, including FOLFIRINOX (5-FU, leucovorin, irinotecan, and oxaliplatin) and gemcitabine combined with nab-paclitaxel, has led to a significant improvement in overall survival compared to treatment with gemcitabine alone [142,143,144]. A recent meta-analysis published in the Cochrane Database of Systematic Reviews (2024) evaluated the effectiveness of chemotherapy and radiotherapy in advanced pancreatic cancer, providing updated evidence on survival outcomes and treatment benefits. The meta-analysis showed that FOLFIRINOX significantly improves OS in advanced pancreatic cancer (HR 0.51), lowering the 12-month mortality risk from 767 to 524 per 1000 patients, and delaying the deterioration of quality of life [143]. Furthermore, combination chemotherapy regimens have demonstrated significant reductions in mortality among patients with advanced pancreatic cancer, as evidenced by high-certainty data showing that FOLFIRINOX reduced the 12-month mortality rate from 76.7% to 52.4% (HR 0.51), gemcitabine plus fluoropyrimidines from 76.7% to 72.2% (HR 0.88), and gemcitabine plus taxanes from 76.7% to 64.4% (HR 0.71) [142].

The integration of neoadjuvant chemotherapy in borderline resectable and locally advanced pancreatic cancer has increased the rates of complete (R0) resections and, together with adjuvant chemotherapy following surgical resection, has significantly improved long-term survival outcomes [143,144,145,146].

## 8. Limitations and Future Directions

This narrative review has several limitations. As it is not a systematic review, the selection of studies may introduce selection bias, and some relevant articles could have been unintentionally omitted. Furthermore, most of the included studies were observational, which may limit the ability to draw causal inferences. Differences in study design, population characteristics, and definitions of early-onset pancreatic cancer also contribute to heterogeneity among the findings. To mitigate these limitations, a comprehensive literature search was performed using multiple keywords, and efforts were made to include data from large, representative cohorts and high-quality registries. Future systematic reviews and meta-analyses are needed to further validate and refine the observations presented here.

## 9. Conclusions

Multiple global and regional data sources consistently show a rising incidence of PC over the past decades, with certain regions—particularly low–middle SDI areas—and younger populations experiencing the most significant increases. While some of this surge may be attributed to better detection, especially for early-stage endocrine cancers, the overall trend underscores an urgent need for enhanced prevention strategies, improved diagnostic techniques, and greater attention to modifiable risk factors such as obesity, diabetes, and tobacco use.

PC is now the third-leading cause of cancer-related death in the U.S., with its mortality rate increasing by 0.3% annually since 2000 and the sixth leading cause of cancer-related deaths worldwide. From 1990 to 2021, global PC deaths more than doubled (from 196,000 to over 505,000), and projections suggest this upward trend will continue. The most dramatic increases have been observed in low–middle SDI and high-SDI regions, where ASMRs have surged.

### 9.1. The Role of Modifiable Risk Factors

The growing body of evidence highlights the central role that modifiable risk factors—particularly obesity—play in driving the rising incidence and mortality of PC. Data show that higher PC rates in men are linked to obesity, alongside smoking, alcohol use, and physical inactivity. In the U.S., 17.9% of PC cases are attributed to excess body weight.

Recent GBD data further emphasize the rise in obesity-related PC deaths, highlighting the urgent need for public health interventions to curb the obesity epidemic and mitigate its impact on cancer outcomes.

### 9.2. Early-Onset Pancreatic Cancer

Recent analyses indicate a significant rise in EOPC, particularly in countries with increasing HDI levels across the Americas, Oceania, and Africa. This rise is strongly linked to the growing prevalence of modifiable risk factors—especially obesity, diabetes, and smoking—among younger populations. This underscores the need for targeted prevention and screening strategies for younger individuals.

Despite these trends, the exact causes behind the increase in EOPC remain unclear, pointing to the urgent need for further research to identify its underlying mechanisms.

### 9.3. The Need for Coordinated Public Health Interventions

The growing burden of PC in both high-income and low-income regions emphasizes the importance of coordinated public health strategies. Projections indicate that the incidence of PC will continue to rise through 2040 and beyond, making it crucial to address the modifiable risk factors driving this trend. By focusing on prevention, early detection, and lifestyle modifications, public health efforts can play a significant role in mitigating the impact of this devastating disease.

### 9.4. Advances in Therapeutic Strategies Leading to Improved Survival

Recent years have seen significant improvements in pancreatic cancer care across surgery, systemic therapy, and care delivery. On the surgical front, innovative techniques—such as artery-first dissection, radical antegrade modular pancreatosplenectomy and refined vascular resection methods—have raised the likelihood of achieving margin-negative (R0) resections and improved postoperative outcomes. Moreover, concentrating patient management in high-volume, specialized centers has yielded substantially better perioperative results (including lower complication and mortality rates), higher survival, and broader access to multimodal therapies. In parallel, perioperative systemic treatments—particularly neoadjuvant chemotherapy and chemoradiotherapy—now frequently downstage borderline resectable or locally advanced tumors, enabling higher surgical resection rates and improved long-term survival. Additionally, the adoption of multi-agent chemotherapy regimens such as FOLFIRINOX and gemcitabine plus nab-paclitaxel has significantly extended overall survival compared to single-agent therapy. Together, these advancements are translating into meaningful progress against this historically aggressive malignancy.
